# Supplementary material for: Potential Contributors to Increased Pulmonary Embolism Hospitalizations During the COVID-19 Pandemic: Insights From the German-Wide Helios Hospital Network
Source: Front Cardiovasc Med. 2021 Aug 12;8:715761. doi: 10.3389/fcvm.2021.715761 (PMC8387932; doi:10.3389/fcvm.2021.715761)

Supplementary Table 1. Total case number and demographics of inpatient cases between May 6 and December 15.

Year	Number of cases	Age (mean ± SD)	Age (Median [IQR])	Females (%)
2016	730,881	55.6 ± 25.4	62 [38, 76]	52
2017	734,302	56.0 ± 25.3	62 [39, 77]	52
2018	726,545	56.2 ± 25.4	62 [39, 77]	52
2019	732,497	56.7 ± 25.2	63 [40, 77]	51
2020	657,296	57.5 ± 24.9	63 [42, 78]	51

Supplementary Table 2. Total and daily admissions with pulmonary embolism between May 6 and December 15.

Year	Total admissions	Daily admissions	CRR (95% CI)	*P* value
2020	2,404	10.7		
2019	2,202	9.8	0.92 (0.86 − 0.97)	< 0.01
2018	2,112	9.4	0.88 (0.83 − 0.93)	< 0.01
2017	2,236	10.0	0.93 (0.88 − 0.99)	0.01
2016	2,167	9.7	0.90 (0.85 − 0.96)	< 0.01

Supplementary Figure 1. Total weekly hospital admissions for pulmonary embolism, non-Covid-19 pneumonia, computed tomography pulmonary angiography, volume depletion and new SARS-CoV-2 infections in Germany (from top to bottom). Smooth curves for weekly admission rates were fitted via Locally Weighted Scatterplot Smoothing (LOESS) with a degree of smoothing of α = 0.2.

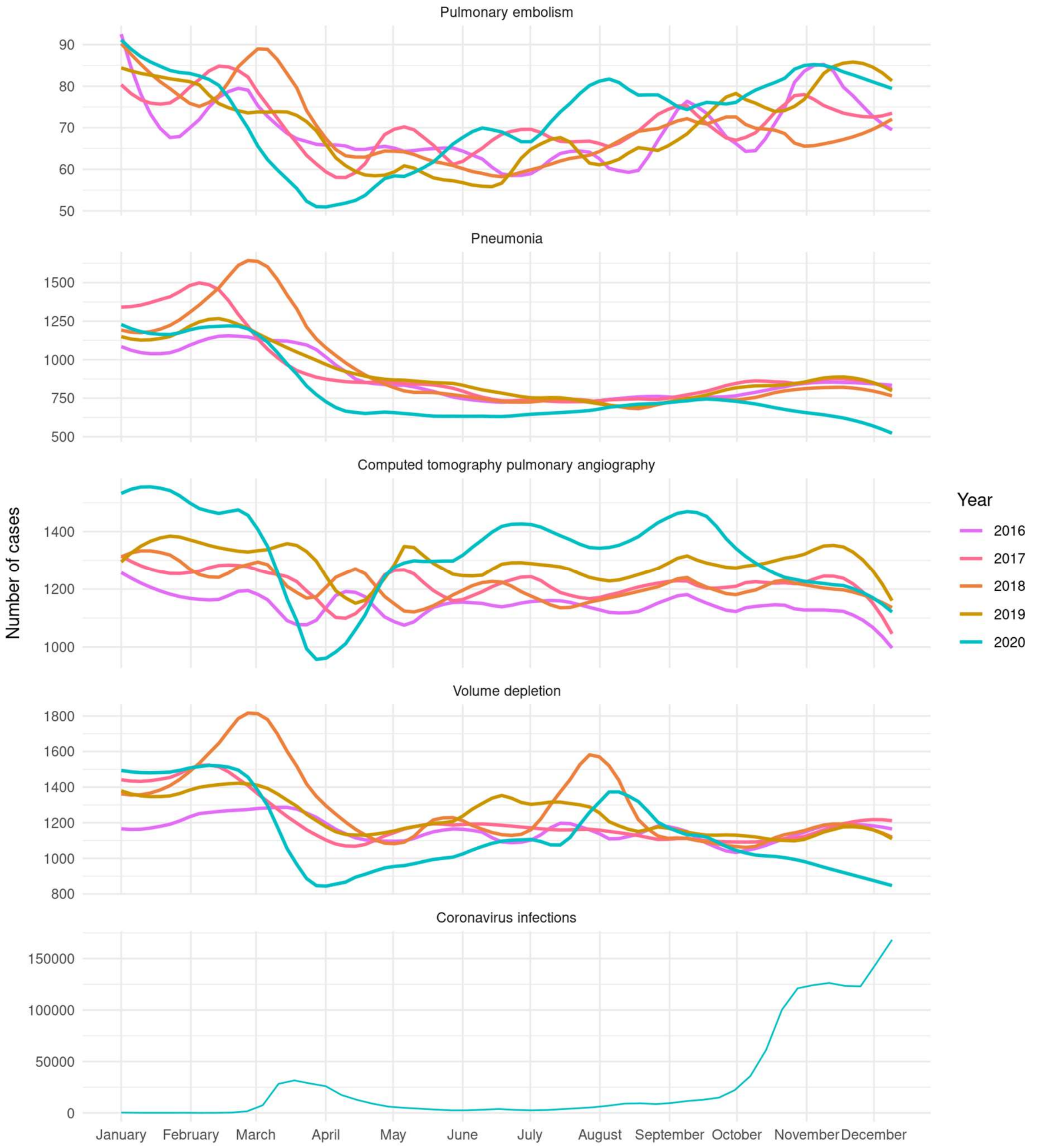

Supplement: Supplementary file 1 [file Data_Sheet_1.pdf]
